# Predicting Factors of Worse Prognosis in COVID-19: Results from a Cross-sectional Study on 52 Inpatients Admitted to the Internal Medicine Department

**DOI:** 10.2174/0118715303288042240111070057

**Published:** 2024-01-18

**Authors:** Giuseppe Lisco, Antonio Giovanni Solimando, Assunta Stragapede, Anna De Tullio, Cristiana Laraspata, Carola Laudadio, Vito Angelo Giagulli, Marcella Prete, Emilio Jirillo, Annalisa Saracino, Vito Racanelli, Vincenzo Triggiani

**Affiliations:** 1 Interdisciplinary Department of Medicine, Section of Internal Medicine, Geriatrics, Endocrinology and Rare Diseases, School of Medicine, University of Bari Aldo Moro, 70124 Bari, Italy;; 2 Guido Baccelli Unit of Internal Medicine, Department of Precision and Regenerative Medicine and Ionian Area-(DiMePRe-J), School of Medicine, Aldo Moro University of Bari, 70124 Bari, Italy;; 3 Operative Unit of Infectious Diseases, Hospital-University Polyclinic of Bari, Bari, Italy;; 4 Centre for Medical Sciences (CISMed), University of Trento, Trento, Italy

**Keywords:** SARS-CoV-2, COVID-19, hypercholesterolemia, free triiodothyronine, interleukin-6, leukocytosis, predicting factors

## Abstract

**Background:**

The initial phases of the COVID-19 pandemic posed a real need for clinicians to identify patients at risk of poor prognosis as soon as possible after hospital admission.

**Aims:**

The study aimed to assess the role of baseline anamnestic information, clinical parameters, instrumental examination, and serum biomarkers in predicting adverse outcomes of COVID-19 in a hospital setting of Internal Medicine.

**Methods:**

Fifty-two inpatients consecutively admitted to the Unit of Internal Medicine “Baccelli,” Azienda Ospedaliero – Universitaria Policlinico of Bari (February 1 - May 31, 2021) due to confirmed COVID-19 were grouped into two categories based on the specific outcome: good prognosis (n=44), patients discharged at home after the acute phase of the infection; poor prognosis, a composite outcome of deaths and intensive care requirements (n=8). Data were extracted from medical records of patients who provided written informed consent to participate.

**Results:**

The two study groups had similar demographic, anthropometric, clinical, and radiological characteristics. Higher interleukin 6 (IL-6) levels and leucocyte count, and lower free triiodothyronine (fT_3_) levels were found in patients with poor than those with good prognosis. Higher IL-6 levels and leucocyte count, lower fT_3_ concentration, and pre-existing hypercholesterolemia were independent risk factors of poor outcomes in our study population. A predicting risk score, built by assigning one point if fT_3_ < 2 pg/mL, IL-6 >25 pg/mL, and leucocyte count >7,000 n/mm^3^, revealed that patients totalizing at least 2 points by applying the predicting score had a considerably higher risk of poor prognosis than those scoring <2 points (OR 24.35 (1.32; 448), *p* = 0.03). The weight of pre-existing hypercholesterolemia did not change the risk estimation.

**Conclusion:**

Four specific baseline variables, one anamnestic (pre-existing hypercholesterolemia) and three laboratory parameters (leucocyte count, IL-6, and fT3), were significantly associated with poor prognosis as independent risk factors. To prevent adverse outcomes, the updated 4-point score could be useful in identifying at-risk patients, highlighting the need for specific trials to estimate the safety and efficacy of targeted treatments.

## 1 INTRODUCTION

The severe acute respiratory syndrome coronavirus 2 (SARS-CoV-2) was officially identified as the etiological agent of a novel infectious disease named coronavirus disease 2019 (COVID-19) in December 2019 [1]. Since then, around 670 million people worldwide have been infected, with at least 6.9 million deaths registered, and more than 13 billion doses of COVID-19 vaccines have been administered so far [2]. After three years of the pandemic, the World Health Organization (WHO) declared the end of the global state of emergency, but COVID-19 remains a global threat with potentially seasonal courses and exacerbations [3, 4].

Evidence shows that SARS-CoV-2 infection occurs with different clinical phenotypes resulting in variable and sometimes unpredictable outcomes [5]. Advanced age, male gender, the number and severity of chronic background diseases, and immune system dysfunction were initially identified as the leading individual determinants of worse progression in COVID-19 [6-8]. Environment, demography, and social and political conditions also affected the viral transmission and correlated to COVID-19 prognosis. Population bulk, density, and urbanization accelerated the human-to-human viral spread, increased the number of detected cases, and finally, the number of deaths. Policies promoting tourism, political divisions (especially in democracies), corruption, social inequity and injustice, and low levels of technological savings to provide proper and safe monitoring of the epidemic's spread and progression (*e.g*., contact tracing) [9] also contributed to viral transmission. On the other hand, a high level of education, trust in government, diligent application of laws, and availability of a number of hospital beds and testing kits have been associated with fewer cases and deaths [9].

Nevertheless, more research is needed to better understand the clinical meaning of baseline characteristics in driving clinical progression of COVID-19, and to quickly identify those patients at risk of worse outcomes in a hospital setting. This real-life study aimed to estimate the weight of predicting factors related to worse progression of COVID-19 considering baseline parameters, such as anamnestic information, clinical and instrumental data, serum biomarkers, and hormonal parameters.

## 2 METHODS

### 2.1 Study Design, Ethics Approval, and Institution

An observational prospective study was designed and conducted on 52 inpatients consecutively admitted to the Unit of Internal Medicine “Baccelli,” Azienda Ospedaliero – Universitaria Policlinico of Bari (February 1 - May 31, 2021). The study was conducted in a real-life emergency setting during the so-called third wave of the COVID-19 epidemic in the South of Italy.

Diagnostic and therapeutic procedures were carried out according to updated recommendations, and patients were managed in line with the principles of good clinical practice and in conformity with the ethical principles of the Declaration of Helsinki. The study was approved by the ethical committee of the University of Bari.

### 2.2 Case Definition

Confirmed COVID-19 cases were defined according to the worldwide accepted definition by the WHO, referring to a person with signs and symptoms (*e.g*., fever, cough, evidence of acute respiratory infection) and a positive nucleic acid amplification test on a nasopharyngeal swab [10].

### 2.3 SARS-CoV-2 Strains

The alpha variant (lineage B.1.1.7) was the most prevalent strain of SARS CoV-2 during the study period in Italy accounting for around 75% of cases.

### 2.4 Definition of Prognosis

The prognosis was defined considering the ordinal scale score modified by the WHO, which identifies a 6-point outcomes score [11] by grouping the individuals into two main categories: 1) those not requiring additional care, oxygen support, or ventilation and who have been discharged to home for convalescence (good prognosis); 2) those requiring respiratory support, intensive care, or who have died (poor prognosis).

### 2.5 Study Protocol

This prospective study aimed to assess baseline characteristics (at the first examination) and the primary study outcome was to assess any relevant difference in demographic, clinical, laboratory, instrumental, and hormonal parameters between patients with better prognoses and those experiencing poor prognoses.

The outcome aimed to provide helpful information on predicting factors of worse progression in COVID-19, therefore highlighting those patients requiring prompt and specific medical management at the bedside soon after hospital admission.

All patients provided a written informed consent to participate before entering the study. Data were extracted from medical records and included age, gender, anthropometric data, comorbidities from a direct anamnestic collection, medical and pharmacological history, arterial pressure, heart rate, respiratory rate, transcutaneous oximetry, chest imaging, the ratio between the arterial partial pressure of oxygen (pO_2_) and the oxygen inspired fraction (FiO_2_) also known as the P/F ratio, and laboratory data, including humoral markers of inflammation and hormones.

### 2.6 Inclusion Criteria

Patients aged ≥18 years with confirmed COVID-19 infection and admitted to the Unit of Internal Medicine “G. Baccelli” were included in the study.

### 2.7 Exclusion Criteria

Patients with pre-existing endocrine diseases (*e.g*., background thyroid diseases or dysfunction, autoimmune endocrinopathies, parathyroid diseases or calcium–phosphorus imbalance) and those who could not provide written informed consent to participate were excluded from the study.

### 2.8 Study Outcome

The study outcome was to identify specific anamnestic, clinical, instrumental, and serologic markers predicting an unfavorable outcome in patients admitted to hospital care with confirmed COVID-19 infection.

### 2.9 Handling of Laboratory Samples and Assay Methods

The routine laboratory assessment included a complete blood count, renal and liver function, circulating markers of inflammation, biomarkers of glucose control, and lipids. Venous samples were collected at the bedside after the patients’ admission and conveyed to the central laboratory.

All patients were tested for SARS-CoV-2 positivity on nasopharyngeal samples soon after admission to the emergency care unit regardless of the cause of access to care. Therefore, each patient in our study underwent a nasopharyngeal swab; samples were collected and conveyed to the Laboratory of the Unit of Hygiene of the Interdisciplinary Department of Medicine at the University of Bari. Samples were analyzed by real-time reverse transcription polymerase chain reaction (rRT-PCR) test for the qualitative detection of ribonucleic acid from SARS-CoV-2.

Additional samples were collected and conveyed to the Laboratory of Endocrinology for hormonal measurements. Serum adrenocorticotropic hormone (ACTH) was measured by a solid phase two-site immunoradiometric assay (ELSA-ACTH, Cisbio Bioassay model 21, 2013, France) with a within-run imprecision ≤6.1% and between-run imprecision ≤5.3%. Serum cortisol was measured by a competitive radioimmunological method (Cortisol RIA Kit, Beckman Coulter, Czech Republic, ref IM1841) with a within-run imprecision ≤5.8% and between-run imprecision ≤9%. Serum thyroglobulin (Tg) was measured by an immunoradiometric assay (THYRO, Cisbio Bioassay model 18, 2018, France) with a within-run imprecision ≤7% and a between-run imprecision ≤14.6%. The sex-hormone binding globulin (SHBG) was measured by a direct immunoradiometric assay (SHBG IRMA Kit, IZOTOP Ltd., Hungary, ref. RK-86CT) with an intra-assay imprecision ≤8.6% and inter-assay imprecision ≤6%. Serum parathyroid hormone (PTH), luteinizing hormone (LH), follicle-stimulating hormone (FSH), total testosterone (TT), thyroid-stimulating hormone (TSH), free thyroxine (fT4), free triiodothyronine (fT3), anti-thyroglobulin antibodies (Ab-Tg), and anti-thyroperoxidase (Ab-TPO) levels were measured by a chemiluminescence method (CLIA, LIAISON XL, DiaSorin Inc., USA).

DiaSorin LIAISON PTH Ref. 310630 was used to measure intact PTH (1-84) with a within-run imprecision ≤7% and between-run imprecision ≤8%. DiaSorin LIAISON LH Ref. 312201 was used to measure LH with a within-run imprecision ≤6.8% and a between-run imprecision ≤9%. DiaSorin LIAISON FSH Ref. 312251 was used to measure FSH with a within-run imprecision ≤5.6% and between-run imprecision ≤4.8%. DiaSorin LIAISON Testosterone xt Ref. 318410 was used to measure TT with a within-run imprecision ≤3.5% and between-run imprecision ≤7.9% (limit of detection 0.01 ng/mL). DiaSorin LIAISON TSH Ref. 311211 was used to measure TSH with a within-run imprecision ≤5.3% and between-run imprecision ≤5.5%. DiaSorin LIAISON FT4 Ref. 311611 was used to measure fT4 with a within-run imprecision ≤2.4% and between-run imprecision ≤4.8%. DiaSorin LIAISON FT3 Ref. 311531 was used to measure fT3 with a within-run imprecision ≤4.7% and between-run imprecision ≤4.7% (limit of detection 1 pg/mL). DiaSorin LIAISON anti-Tg Ref. 311711 was used to measure Ab-Tg with an intra-assay imprecision ≤3.2% and inter-assay imprecision ≤8.9%. DiaSorin LIAISON anti-TPO Ref. 311701 was used to measure Ab-TPO with an intra-assay imprecision ≤6.2% and inter-assay imprecision ≤6.6%.

### 2.10 Statistical Analyses

The results have been expressed in the form of descriptive statistics aiming to provide a better understanding of clinical and laboratory variables significantly affecting the prognosis of the examined patients.

Patients were split into two groups based on the prognosis. The first group included survivors (n=44) who concluded the hospital stay without experiencing any complications and were regularly discharged at home to recover after the acute phase of the infection. The second group included patients (n=8) with a worse prognosis, a composite outcome of intensive care requirement, and death occurring during hospital stay.

Continuous variables have been expressed as mean and standard deviation or median and interquartile range according to their distribution. Gaussian (or normal) distribution of continuous variables was verified by the Shapiro-Wilk test, while the variance equality was tested with the F-test. Categorical variables have been expressed as frequency with numbers and percentages. The two-tail unpaired T-test was used to calculate the mean difference between normally distributed variables. In contrast, the difference between non-normally distributed variables was calculated using non-parametric tests (*e.g*., Mann-Whitney U-test). The difference in terms of frequencies and risk or odds ratios between categorical variables was assessed by the Fisher’s test.

Generalized linear models were used to conduct regression analysis and analysis of variance for multiple dependent variables employing one independent variable (prognosis) with the aim of testing for potential predicting factors negatively affecting the primary outcome. The statistical significance level was set to a *p*-value <.05.

## 3 RESULTS

Fifty-two (16 women and 36 men) patients were consecutively included in the study. The age of participants ranged from 38 to 80 years. The median time of hospital stay was 12 days, ranging from 0 to 40. Among 52 inpatients, 3 (6%) were referred to the intensive care unit because of relevant deterioration of clinical conditions, and 5 (9%) died.

COVID-19 diagnoses were confirmed 24 hours before the hospital admission in 10 patients. Forty-two patients received a confirmed diagnosis from 2 to 71 days before the hospital admission. More precisely, 8 had a diagnosis four weeks before and were therefore admitted to hospital care because of signs and symptoms of long-COVID. The diagnosis of COVID-19 was confirmed after the hospital admission only in one patient (5 days later) who was admitted to hospital care due to signs and symptoms highly suspicious of COVID-19.

Signs and symptoms of COVID-19 occurred 1 to 23 days before the confirmation of cases in 11 (21%), concomitantly in 9 (17%), and after (1 to 32 days later) in 28 (54%). Data were unavailable or inconsistent for 4 patients (8%). The most common clinical presentation of COVID-19 included respiratory signs and symptoms (cough and dyspnea), fever, and febrile-related symptoms. Almost all (49 out of 52) had radiological findings of pulmonary consolidation, the leading reason for admission to the Internal Medicine department.

The mean time of viral clearance, defined as the number of days across the confirmation of a case to the first negative nasopharyngeal swab, was 30 days.

The background characteristics of the study population are shown in Table **1**. Inpatients were mostly men (78%), with a median age of 61. Comparisons between the two study groups revealed that the male gender was more frequently associated with worse clinical progression of COVID-19 and, even if the result was not statistically significant, women were less likely to experience poor prognosis compared to men (odds ratio or OR = 0.28; CI 95% (0.006; 2.5)). Clinical, anthropometric, and laboratory parameters were statistically similar between the two groups apart from the free triiodothyronine (fT_3_) level, leucocyte count, and interleukin 6 (IL-6). More precisely, fT_3_ was lower (1.57 (0.41) *vs*. 1.99 (0.48); *p* =0.026), and IL-6 (42.2 (190.5) *vs*. 10.4 (22.5); *p* =0.047) and leukocyte count (10,700 (7,735) *vs*. 6,240 (4,297,5); *p* =0,0021) were higher in patients with poor prognosis compared to those with good prognosis. A relevant change in the leucocyte formula of patients with worse compared to those with better prognosis was also observed with increased neutrophil-to-lymphocyte ratio among the former. However, the result was not statistically significant (Table **1**).

**Fig. (1):**
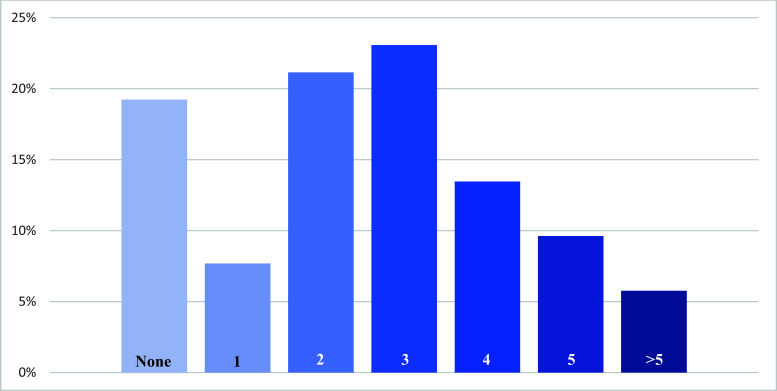
Distribution of the number of background comorbidities in the study population. **Note:** The graphic depicts the distribution, in order of the observed frequency, of the number of chronic comorbidities of patients: no comorbidities, 19%; one comorbidity, 8%; two comorbidities, 21%; three comorbidities, 23%; four comorbidities, 13%; five comorbidities, 10%; more than five comorbidities, 6%.

Chronic concomitant diseases of patients ranged from 0 to 7. More precisely, 19% had no established chronic comorbidities or were not taking specific drugs, while most (59%) had 2 to 4 concomitant chronic diseases (Fig. **1**). The most relevant chronic comorbidities were arterial hypertension (29; 56%), type 2 diabetes mellitus (T2D 16; 31%), established atherosclerotic cardiovascular disease (14; 27%), hypercholesterolemia (11; 21%), and obesity (10, 19%).

All patients had undergone baseline chest imaging. Most had radiological findings of pulmonary consolidations with monolateral (12) or bilateral (37) chest involvement. The remaining 3 patients had negative chest imaging (Table **2**). Bilateral pneumonia, suggesting a relevant pulmonary involvement in COVID-19, was initially diagnosed in 6 out of 8 patients (75%) who had undergone COVID-19-related worse progression and 31 of 44 patients (70%) who completely recovered during the hospital stay.

Although the severity of pulmonary involvement could have suggested a more critical baseline status of patients with COVID-19, no relationship was found between the seriousness of chest involvement and the risk of worse progression (Table **2**).

The weight of the most frequently observed background diseases in driving poor prognosis was also calculated. Only those patients with pre-existing hypercholesterolemia had a statistically relevant increase in the risk of poor prognosis upon the infection occurrence (OR 5.28, *p* = .04). No additional risk was found for the other relevant chronic comorbidities. A detailed description of the distribution of cases and risks, estimated as odds ratios, is shown in Table **3**.

Preliminary analyses showed circulating levels of specific serological biomarkers to be significantly different at baseline, although demographics, anthropometric, anamnestic, and clinical characteristics were similar between the two study groups. These biomarkers included the fT_3_, leucocyte count, and IL-6 levels (Figs. **S1**-**S3**). To better understand the role of such biomarkers in predicting unfavorable outcomes (admission to intensive care or death), univariate and multivariate analyses were carried out. Two separate models (patterns 1 and 2) were included in the logistic regression analysis. In pattern 1, three non-modifiable variables with established confounding effects on the prognosis were included (gender, qualitative dichotomic variable (m/f); age, continue variable (yrs); comorbidities, discrete countable variable (n)). As detailed in Table **4**, baseline leucocyte count (0.025 ± 0.009, *p* = 0.009) and IL-6 levels (0.002 ± 0.0004, *p* = ~0) positively correlated with poor prognosis. Baseline fT_3_ levels were inversely related to the negative outcome (-0.188 ± 0.07, *p* = 0.016). Given the weight of established diagnosis of hypercholesterolemia, but not the levels of lipid control, in potentially driven poor prognosis (Table **3**), the pattern 2 multivariate analysis included each disease considered as comorbidity (qualitative dichotomic variables (y/n)). After this adjustment, the overall result did not change significantly but confirmed pre-existing hypercholesterolemia to be another independent risk factor associated with poor outcomes (Table **5**).

To summarize our findings, a predicting score to predict inpatients' clinical course of COVID-19 was designed. The three laboratory variables of interest were included in the score. More precisely, one point to each of the following conditions was assigned: fT_3_ levels < 2 pg/mL, IL-6 levels >25 pg/mL, and leucocyte count >7,000 n/mm^3^. The score ranged from 0 to 3 points. Pearson's Chi-squared test was then conducted to assess the association between the clinical outcome and the above score, revealing a statistically relevant positive relationship between the two variables (Table **6**).

Patients who experienced poor prognoses totalized at least 2 points after applying the predicting score while stratifying the risk. Therefore, an additional analysis was carried out to estimate better the chance of worse progression by comparing high-risk (≥2 points) to low-risk patients (<2 points). The risk estimation confirmed that patients totalizing at least 2 points by applying the predicting score had a considerably higher risk of poor prognosis than those with a score <2 points (Table **7**; relative risk: 17; OR 24.35, *p* =.03).

After adjusting the pathological score by including the variable hypercholesterolemia, only four patients passed from score 1 to score 2 and were upgraded to the positive score (2 or more points). Nevertheless, the results did not change significantly after this adjustment.

## 4 DISCUSSION

Managing acute and chronic diseases during the first waves of the COVID-19 pandemic was significantly affected by a broad and unprecedented reorganization of healthcare facilities and work overload. Potential pitfalls in the in-hospital management of COVID-19 patients could have been, at least in part, the consequences of a sudden difficult-to-cope experience. Reliable predicting scores are desirable to improve the quality of care and better cope with similar difficulties in the future.

The novelty of this study is in the aim. The baseline assessment is an essential element of patient-centered care at the bedside; however, the weight of every baseline anamnestic, clinical, and laboratory parameter to predict the short-term (in-hospital) prognosis has yet to be entirely understood in COVID-19 patients. Moreover, since evidence has been provided on the bidirectional relationship between the endocrine system and COVID-19, this study’s novelty was in the inclusion of some hormonal parameters among latent predicting factors of poor prognosis in hospitalized patients.

### 4.1 Baseline Characteristics in the Prediction of Worse Prognosis

Our results showed baseline characteristics of examined patients to not be dissimilar between those experiencing positive and negative clinical courses or prognoses, even if some specific differences were found. First, a high frequency of men (79%) was observed in both groups (discharged: 66% *vs*. worse prognosis: 90%). The results aligned with other reports, suggesting that men were more prone to be hospitalized, and experienced worse progression and death in case of COVID-19. The mechanisms explaining this relationship highlighted a gender medicine issue, and several pathophysiological hypotheses were provided to illustrate the burden. More emphasis was given to background chronic comorbidities, general health status, and attitude to follow hygienic tips properly [12]. Nevertheless, hormonal background dichotomy between the two genders should also be considered to explain the difference in terms of outcomes. Less immune system efficiency, more significant viral load and shedding, slower viral clearance, more extended pulmonary and extra-pulmonary inflammation, endothelial dysfunction, and thrombophilia were hypothesized as the basis of poor prognosis in men compared to women [13].

Second, apart from a shorter hospital stay of patients experiencing poor prognosis compared to the others, which is the obvious consequence of different outcomes, no specific dissimilarity in anthropometric and clinical parameters was found between the two groups. As an example, arterial pressure, heart rate, body temperature, respiratory rate, and transcutaneous oximetry were comparable between the two study groups suggesting that some of the easy-to-check parameters, which the general population was advised to monitor properly at home in case of signs and symptoms of SARS-CoV-2 infection, could not be well-representative of risks related to poor outcomes in patients hospitalized due to COVID-19. Nevertheless, these parameters could be affected significantly by background treatments, such as antipyretics or oxygen in previous steps of care (*i.e*., emergency care unit). Therefore, caution should be considered while interpreting this result. However, the P/F ratio, a reliable marker of pulmonary performance, was also similar between the two groups, and its mean value was higher than 300 in all patients. It is well-established that 300 is a sensitive and specific cut-off point for the definition of respiratory failure in acute pulmonary injury. Overall, the results suggest that the lack of functional pulmonary impairment at baseline could not exclude the possibility of short-term deterioration of prognosis in COVID-19. Similarly, baseline chest imaging (CT scan for most) suggesting the presence of mono- or bilateral radiological signs of pneumonia was a common finding in both groups without any relevant difference.

Third, advanced age was one of the most important non-modifiable risk factors of poor prognosis in COVID-19 patients [14]. Poor health, background comorbidities, overall frailty, and high susceptibility to further complications after hospital admission were other relevant risk factors [15]. Nevertheless, throughout the pandemic evolution, the mean age of infected and hospitalized patients reduced significantly, suggesting that the pass to new phases of the epidemic was characterized by faster progression of viral transmission across the entire population, therefore involving younger individuals. This phenomenon was evident in our study, in which the mean age of patients was around 60 years, thus two decades lower compared to that observed ten months before [14]. Hence, the age gap as the leading determinant of poor prognosis disappeared, leading us to consider other prognostic factors. Once again, the burden of comorbidity and poor baseline health were hypothesized as predisposing factors to poor prognosis. Our data found no statistically relevant difference in the number of background comorbidities between the two groups. However, in a secondary analysis carried out after splitting every single comorbidity to weigh the risks better, we found that the chance of poor outcomes was 5 times higher in patients with pre-existing hypercholesterolemia than those without. According to a meta-analysis, pre-existing dyslipidemia (mostly hypercholesterolemia) was associated with a 60% increase in short-term mortality risk of COVID-19 patients [16]. Other data have confirmed dysmetabolic patients, including those with hypercholesterolemia, to be more prone to acute complications, negatively affecting the COVID-19 prognosis upon the infection occurrence [17, 18] or death [19]. The lipid profile, expressed by the measurement of serum lipids and some related lipoproteins, showed no specific difference between the two study groups at baseline. Hence, our data did not find similar results as described elsewhere. During the acute phase of the infection, the levels of LDL and HDL cholesterol and apolipoprotein A-I, A-II, and B were reduced [20]. These changes were described as usually transient, and the lipid profile inclined toward baseline values or deteriorated, possibly predisposing non-hypercholesterolemic patients to hypercholesterolemia during and after the convalescence. Patients with long-lasting reduced HDL cholesterol and apolipoprotein A-I were considered at risk of poor prognosis due to cardiovascular complaints during COVID-19 [20]. In our study, HDL cholesterol levels were lower than expected in most patients, but no difference was found between the two groups. We can speculate that patients who experienced poor prognoses had a heavier background cardiovascular risk and should have reached more stringent lipid targets than observed. However, due to the nature of our study, no information about lipid trends over time has been made available. Some hypotheses have been proposed better to understand the relationship between hypercholesterolemia and COVID-related mortality. First, hypercholesterolemia per se may increase the risk of incident arterial and venous thromboembolism, as demonstrated in patients with heterozygosis familial hypercholesterolemia, who showed an increased incidence of CV events after SARS-CoV-2 infection [21]. Moreover, the in-hospital administration of anti-hypercholesterolemic medications, primarily statins, was found to reduce the risk of poor outcomes in COVID-19, probably due to additional protective mechanisms, other than the cholesterol-lowering effect, such as a direct cardio-protective effect and anti-oxidative properties [22, 23].

Fourth, diabetes, the level of glucose control (*e.g*., HbA1c), and related comorbidities (such as obesity) were not associated with poor prognosis as hypothesized [24] and demonstrated [25] before. Other reports have found patients with metabolic syndrome to be more prone to severe COVID-19 [26], indicating that mild hyperglycemia and pre-diabetes, in addition to other CV risk factors, including overweight obesity, hypertriglyceridemia, and low levels of HDL cholesterol as some examples, may affect the prognosis upon SARS-CoV-2 infection occurrence. However, a recent paper reporting joint data from Ontario and Copenhagen suggested diabetes mellitus per se to not be associated with a high mortality risk among hospitalized COVID-19 patients [27]. Besides relevant differences in study populations, study designs, number of events, and clinical outcomes, glucose management of COVID-19 patients may affect the risk. As preliminary evidence suggested that poorly controlled diabetes mellitus should be considered a relevant risk factor predisposing to poor outcomes in COVID-19, the management of glucose levels improved remarkably soon after the first wave of the pandemic. In addition, each specific anti-hyperglycemic medication could have the potential for both benefits and harms in COVID-19 patients [24]. As some examples, the use of metformin and glucagon-like peptide 1 receptor agonists was associated with better prognosis while administered to treat hyperglycemia in hospitalized patients with COVID-19 [28]. Similar protection was also confirmed in T2D patients with preadmission use of gliflozins [29, 30] and oral gliptins [31]. Conversely, some concerns have been raised for insulin users, as in-hospital insulin treatment was associated with poor outcomes, especially in aged people [32, 33]. In our population, 16 patients had established T2D and achieved optimal glucose control. Most of them continued taking pre-admission oral medications. At the same time, composite insulin regimens were introduced shortly after the deterioration of glucose control in a few individuals, especially those with clinical and laboratory signs of severe sepsis. Nevertheless, due to the exiguity of the number of events, carrying out specific sub-analyses was not convenient, and the discussion mentioned before is speculative.

### 4.2 Baseline Biochemical and Hormonal Markers in the Prediction of Worse Prognosis

Inflammatory markers are reliable features to diagnose and classify the severity of COVID-19. In our study, patients who experienced poor prognoses had higher background levels of C-reactive protein, leucocyte count, neutrophil-to-lymphocyte ratio, IL-6 level, and ferritin. However, leucocyte count and IL-6 were only significantly higher in patients with poor than favorable prognoses. Our data are in line with other studies assessing the role of inflammatory biomarkers as predicting factors of COVID-19 severity [34-36] and confirm the importance of such biomarkers in the pathophysiology of COVID-19 and its related sequelae, including the post-COVID or long-COVID syndrome [37, 38]. No electrolyte imbalances were found in our study, including calcium, magnesium, and phosphorous homeostasis. So, our data did not confirm previous evidence suggesting the role of hypocalcemia [39] and vitamin D deficiency/insufficiency as potential markers of poor prognosis in hospitalized patients with COVID-19 [40-42]. Hypocalcemia has been observed in critical conditions, including severe COVID-19, and some specific mechanisms underlying its pathophysiology have been outlined. These include higher levels of procalcitonin, which are usually elevated in most severe bacterial and even not-bacterial sepsis, and hypoalbuminemia, suggesting the latter to be due to pre-existing poor nutrition, poor health, or the presence of concomitant and extensive renal or liver injury [43].

The SARS-CoV-2 entry into the cell is mediated by some specific and obligate receptors, such as the angiotensin-converting enzyme (ACE) receptor, transmembrane protease serine 2 (TMPRSS2), cathepsin L, and furin [44]. Even if the concentration of these receptors differs considerably across the human tissues, their distribution is almost ubiquitarian, and the endocrine system could be affected by SARS-CoV-2 upon the infection occurrence [45]. Subacute and silent thyroiditis have been previously described as the consequence of SARS-CoV-2 infection and new-onset and relapsing autoimmune thyroid diseases [45]. Our study found patients experiencing poor prognoses to have lower levels of TSH, circulating thyroglobulin, and fT_3_, even if the results only reached the statistical significance for the latter variable. Most importantly, as per protocol, patients with pre-existing thyroid dysfunction were excluded, and this was certified by the absence of signs of thyroid autoimmunity, including TSH receptor antibodies, and the fact that none were taking levothyroxine replacement or anti-thyroid drugs. Our data aligned with the results of another study on 191 patients with mild-to-moderate (severe cases only in 3%) COVID-19 that found that 15% of patients exhibited thyroid dysfunction phenotypically characterized by a reduction in TSH and fT_3_ levels. The study also confirmed that the more the severity of COVID-19, the lower the values of circulating fT_3_ [46], as also suggested by other authors [47-49]. The so-called euthyroid sick syndrome, or the low T3 syndrome, is characterized by laboratory evidence of normal or lower than normal TSH values, with reduced levels of fT_3_ and variable fT_4_ values in the absence of acute and chronic diseases affecting the hypothalamus-pituitary-thyroid axis. As per definition, the euthyroid sick syndrome is a functional disturbance manifesting concomitantly to several disorders, including malnutrition, poor health and frailty, advanced chronic diseases, and sepsis [50, 51]. The pathophysiology of euthyroid sick syndrome is complex and multifactorial [52]. Proinflammatory cytokines, such as the IL-6, play a role in suppressing the hypothalamic synthesis and release of the TSH-releasing hormone, which stimulates the pituitary thyrotropic cells to release TSH and blunt the TSH release directly. Inflammatory cytokines reduce the synthesis of the peripheral type 1 deiodinase, which in turn is responsible for the tissue conversion of circulating thyroxine to fT_3_. This is believed to be the primary mechanism explaining the reduced levels of fT_3_ in sepsis and severe immune-inflammatory disorders. Moreover, high cortisol levels, loss of appetite, and hypo-nutrition also affect peripheral thyroid hormones’ metabolism and transport. Indeed, synthetic and adrenal-derived glucocorticoids negatively modulate the synthesis of peripheral type 1 deiodinase and circulating thyroid hormone-binding globulin, which is responsible for the transport of thyroid hormones from the thyroid to peripheral tissues. The latter phenomenon is also conditioned by the concomitant increase in circulating levels of not-esterified free-fatty acids due to insulin resistance and malnutrition during severe acute illness. Most importantly, we found the levels of fT_3_ to be inversely related to the risk of poor prognosis independently compared to the levels of IL-6. This is an important finding suggesting that, although higher levels of IL-6 play a role in dropping circulating levels of fT_3_, the concomitant occurrence of low circulating fT_3_ and elevated IL-6 levels may have a synergistic effect on the deterioration of prognosis in COVID-19 [53]. This hypothesis is supported by a potential bidirectional relationship existing between fT_3_, and IL-6 levels. Indeed, in experimental conditions, the thyroid hormone receptor agonism in the liver blunted the hepatic IL-6 signaling during endotoxemia, indicating that sufficient levels of circulating fT_3_ have the potential to turn off the systemic inflammation cascade by acting at a crucial point of the inflammatory machinery [54]. In survivors, thyroid function changes have been found to be less evident and described as transient, with most patients recovering background thyroid function after completing the convalesce [55]. This phenomenon suggests that thyroid function changes are attributable to a detrimental interaction among the SARS-CoV-2 infection, systemic inflammation, and the pituitary-thyroid axis, and describes a specific hallmark of severe and critical COVID-19 cases putting an emphasis on the management of those patients.

Men exhibited lower than expected serum TT and SHBG levels in both groups [56], indicating that COVID-19, systemic inflammation, and certain medications, such as glucocorticoids, may hamper acutely testosterone secretion and transport, leading to male hypogonadism in all. However, no specific difference in the pituitary-testicle axis was found. Our results have also confirmed a relevant involvement of the pituitary-testicular axis in COVID-19 [57-60], even if the drastic reduction of serum TT did not apparently affect the prognosis. On the contrary, our findings did not confirm previous results indicating that low levels of TT may have a role in driving poor prognosis in COVID-19 patients with extensive pulmonary involvement [61]. Dissimilarity in background characteristics of the two study populations could explain the different results.

### 4.3 Predicting Score

Our findings have suggested four specific baseline variables, one anamnestic (pre-existing hypercholesterolemia) and three laboratory parameters (leucocyte count, IL-6, and fT_3_), to be significantly associated with poor prognosis as independent risk factors. To simplify the comprehension of these results, we have elaborated an updated 4-point score (0 to 3), assigning one point to each of the following conditions: fT_3_ < 2 pg/mL, indicating markedly low levels of fT_3_; IL-6 >25 pg/mL [62]; leucocyte count >7,000 n/mm^3^, the median value in our study population. Adding the variable hypercholesteremia did not modify the risk estimation, and the score effectively predicted worse outcomes, especially when at least two of the three criteria mentioned above were evident at baseline (risk ratio 17; OR 24.3). Our findings suggest that individuals with scores equal to or greater than 2 points could have probably been susceptible to specific treatments (IL-6 antagonism [63], triiodothyronine) to prevent the worst outcome, hence indicating the need for more mechanistic and intervention studies.

## CONCLUSION

In conclusion, the study findings provide updated information on the prognosis of patients admitted to an Internal Medicine ward during the third wave of the COVID-19 pandemic in a tertiary care centre at the University of Bari [64]. In that context, some specific biomarkers, namely leukocyte count, fT_3_, and IL-6, have been found to be independent risk factors of worse prognosis in patients with COVID-19 admitted to the Internal Medicine department.

## STRENGTH AND LIMITATIONS

The study has some strengths and limitations. The strength is in the study design and the reliability of the assay methods. In addition, the study provides information on a specific setting of care of Internal Medicine for patients admitted with initially moderate COVID-19 [64], for which an adverse outcome would not have been easily predicted. While the study provides valuable insights also into the potential impact of COVID-19 on the endocrine system, it is important to acknowledge some limitations. The relatively small sample size due to a short window of observation and the single-center study design might have reduced the statistical power of the analyses and limited the generalizability of the findings. However, it should be considered that the study was carried out in the presence of a shortage of available resources, as well as the scenario of challenges in conducting research during the pandemic.

## Figures and Tables

**Table 1:** Baseline characteristics of the study population.

**Variables**	**Discharged (n=44)**	Poor Prognosis (n=8)*	*p*-value**
Men, n (%)	29 (65.9%)	7 (90%)	0.06***
Age (yrs), mean (SD)	61.8 (9.8)	60.5 (10.9)	0.75
Hospital stay (days), mean (SD)	13.1 (6.8)	5.7 (4.2)	0.001
Body weight (kg), mean (SD)	80.2 (13.5)	79.2 (14.7)	0.89
BMI (kg/m^2^), mean (SD)	28.4 (4.9)	27.7 (3.6)	0.51
P/F, mean (SD)	303.1 (103)	332.7 (158.1)	0.62
Serum albumin (g/dL), mean (SD)	3.35 (0.48)	3.06 (0.46)	0.1
Total cholesterol (mg/dL), mean (SD)	162.9 (45.1)	170.1 (39.6)	0.67
LDL cholesterol (mg/dL), mean (SD)	118.2 (40.5)	123.1 (27)	0.69
Free triiodothyronine (pg/mL), mean (SD)	1.99 (0.48)	1.57 (0.41)	0.026
Comorbidities (n), median (IQR)	2 (3)	3 (1)	0.77^#^
Systolic blood pressure (mmHg), median (IQR)	130 (23.2)	127 (20)	0.74^#^
Diastolic blood pressure (mmHg), median (IQR)	80 (12.5)	77.5 (10)	0.49^#^
Heart rate (bpm), median (IQR)	80 (15.2)	77.5 (29)	0.57^#^
Temperature (^o^C), median (IQR)	36.8 (0.6)	36.8 (0.5)	0.9^#^
Respiratory rate (bpm), median (IQR)	18 (4)	19 (3.5)	0.21^#^
Transcutaneous oximetry (%), median (IQR)	96 (2.2)	96.5 (2.5)	0.59^#^
CRP (mg/dL), median (IQR)	36.5 (68.6)	79.4 (38.3)	0.09^#^
D-dimer (mg/dL), median (IQR)	765 (786.7)	604 (2,705.5)	0.9^#^
Leucocyte count (n/mm^3^), median (IQR)	6,240 (4,297.5)	10,700 (7,735)	0.0021^#^
NLR, median (IQR)	3.7 (4)	9.8 (9.6)	0.14^#^
Ferritin (mg/dL), median (IQR)	414.5 (582.2)	616 (370.2)	0.76^#^
IL-6 (pg/mL), median (IQR)	10.4 (22.5)	42.2 (190.5)	0.047^#^
FPG (mg/dL), median (IQR)	107 (68.7)	105 (21.5)	0.56^#^
HbA1c (mmol/mol), median (IQR)	43 (15)	34 (6)	0.09^#^
Triglycerides (mg/dL), median (IQR)	128 (65)	116 (84)	0.59^#^
HDL cholesterol (mg/dL), median (IQR)	36.5 (32)	34 (15.5)	0.9^#^
Creatinine (mg/dL), median (IQR)	0.8 (0.2)	1 (0.4)	0.17^#^
Total testosterone (ng/mL), median (IQR)	0.75 (0.64)	0.47 (0.74)	0.21^#^
LH (U/L), median (IQR)	3.85 (3.5)	3.2 (7)	0.92^#^
FSH (U/L), median (IQR)	6.6 (7.5)	7.8 (3.5)	0.83^#^
SHBG (nM/L), median (IQR)	20 (10)	31 (51.8)	0.73^#^
TSH (mUI/mL), median (IQR)	1.2 (1.4)	0.5 (0.6)	0.29^#^
Free thyroxine (pg/mL), median (IQR)	11.8 (3.6)	11.3 (2.7)	0.97^#^
Serum plasma cortisol (ug/dL), median (IQR)	5 (11.8)	20.7 (24.7)	0.07^#^
Thyroglobulin (ng/mL), median (IQR)	10.7 (9.6)	4.6 (1.25)	0.07^#^
TgAb (UI/L), median (IQR)	5.3 (10.3)	5.8 (8.8)	0.89^#^
TPOAb (UI/L), median (IQR)	1.8 (6.9)	1.4 (3.1)	0.61^#^
Total serum calcium (mg/dL), median (IQR)	8.4 (0.9)	8.7 (1.1)	0.45^#^
Serum calcium after correction for albumin (mg/dL), median (IQR)	9.1 (0.84)	9.2 (1.59)	0.27^#^
PTH intact (pg/mL), median (IQR)	26.2 (18.1)	28.7 (28.8)	0.98^#^
25OH-vitamin D (ng/mL), median (IQR)	25 (8)	22 (5)	0.63^#^

**Note:** Baseline characteristics of the study population included demographics, and anthropometric, laboratory, and hormonal parameters. Data were divided into two categories according to the prognosis and compared by proper statistical analyses; *Composite outcome of deaths (5) and admissions to intensive care unit (3); **Statistically significant with alpha error = 5%; ***Fisher's exact test for count data: odds ratio of poor prognosis women over men = 0.28; CI 95% (0.006; 2.5); ^#^Mann-Whitney U test; **Abbreviations:** number = n; interquartile range = IQR, perfusion/ventilation = P/F; low-density lipoprotein = LDL, C-reactive protein = CRP, neutrophil-to-lymphocytes ratio = NLR, interleukin-6 = IL6, fasting plasma glucose = FPG, glycated hemoglobin = HbA1c, luteinizing hormone = LH, follicle-stimulating hormone = FSH, sex-hormone binding globulin = SHBG, thyroid-stimulating hormone = TSH, anti-thyroglobulin antibodies = TgAb; anti-thyroperoxidase antibodies = TPOAb, parathyroid hormone = PTH.

**Table 2:** Baseline chest radiological findings***.

**Findings**	**Discharged (n=44)**	Poor Prognosis (n=8)*	*p*-value**
No chest involvement	2	1	0.39
Monolater consolidation	11	1	
Bilateral consolidation	31	6	

**Note:** *Composite outcome of deaths (5) and admissions to intensive care unit (3); **Statistically significant with alpha error = 5%; ***Fisher's exact test for count data.

**Table 3:** Estimation of the weight, expressed as odds ratio, of each relevant chronic comorbidity with respect to the individual prognosis.

**Clinical Outcome**				
**Chronic Diseases**	**Discharged** **(n = 44)**	Poor Prognosis* **(n=8)**	**ORs (95%CI)**	*p*-value**	
Hypercholesterolemia (y/n)	7/37	4/4	5.28 (1.06; 26.29)	0.042	
Arterial hypertension (y/n)	25/19	4/4	0.76 (0.17; 3.43)	0.72	
Diabetes mellitus (y/n)	14/30	2/6	0.71 (0.13; 3.99)	0.70	
Established CVD (y/n)	13/31	1/7	0.34 (0.04; 3.05)	0.96	
Obesity (y/n)	8/36	2/6	1.5 (0.25; 8.84)	0.65	

**Note:** The table highlights the difference in the observed frequency of the leading chronic comorbidities (hypercholesterolemia, arterial hypertension, diabetes mellitus, established CVD, and obesity). Odds ratios (ORs) and 95% CI were calculated; *Composite outcome of death (5) and admission to intensive care unit (3); **Statistically significant with alpha error = 5%; **Abbreviation:** CVD = cardiovascular disease.

**Table 4:** Predicting factors of poor prognosis as assessed by a logistic regression model (pattern 1).

**-**	**Estimate**	**Standard** **Error**	**t-value**	***p*-value**
Intercept	-0.021	0.37	-0.06	0.95
Gender (m/f)	0.108	0.08	1.35	0.19
Age (yrs)	0.001	0.004	0.27	0.78
Background comorbidities (n)	-0.011	0.02	-0.57	0.57
Leucocyte count (n/mm^3^)	0.025	0.009	2.76	0.009**
IL-6 (pg/mL)	0.002	0.0004	4.28	0.000013***
fT3 (pg/mL)	-0.188	0.07	-2.52	0.016*

**Note:** Pattern 1 included three non-modifiable confounders: gender, age, and the number of pre-existing comorbidities. Codifying the level of statistical significance (decreasing order): 0 ‘***’; 0.001 ‘**’; 0.01 ‘*’; 0.05 ‘^.^’; **Abbreviations:** IL-6 = interleukin 6; fT_3_ = free triiodothyronine.

**Table 5:** Predicting factors of worse prognosis as assessed by a logistic regression model (pattern 2).

-	**Estimate**	**Standard Error**	**t-value**	***p*-value**
Intercept	-0.201	0.38	-0.52	0.60
Gender (m/f)	0.10	0.07	1.33	0.19
Age (yrs)	0.004	0.004	0.94	0.35
Arterial hypertension (y/n)	-0.141	0.07	-1.95	0.06
Hypercholesterolemia (y/n)	0.324	0.11	2.97	0.005**
Diabetes mellitus (y/n)	-0.006	0.08	-0.07	0.94
Obesity (y/n)	0.0006	0.09	0.007	0.99
Established CVD (y/n)	-0.059	0.09	-0.59	0.56
Leucocyte count (n/mm^3^)	0.026	0.009	2.94	0.006 **
IL-6 (pg/mL)	0.002	0.0005	3.58	0.001**
fT3 (pg/mL)	-0.167	0.07	-2.29	0.029 *

**Note:** Pattern 2 included every single pre-existing comorbidity in addition to gender and age; Codifying the level of statistical significance (decreasing order): 0 ‘***’; 0.001 ‘**’; 0.01 ‘*’; 0.05 ‘^.^’; **Abbreviations:** IL-6 = interleukin-6; fT_3_ = free triiodothyronine.

**Table 6:** Distribution of baseline individual predicting score in relationship with the clinical outcome.

**Predicting Score**					
Clinical outcome	0	1	2	3	*p*-value = 0.005*
Discharged (n)	4	22	14	4	
Poor prognosis (n)**	0	0	4	4	
Accuracy (%)	100	100	50	50	-

**Note:** * Statistically significant with alpha error = 5%; ** Poor prognosis = composite outcome of death (5) and admission to intensive care unit (3).

**Table 7:** Risk estimation of poor prognosis as assessed by dichotomizing the predicting score.

**Predicting Score**				
**Clinical Outcome**	Negative (<2)	**Positive (≥2)**	**OR (95%CI)**	*p*-value*
Discharged (n)	26	18	24.35 (1.32; 448)	0.03
Poor prognosis (n)**	0	8		
Accuracy (%)	100	100		

**Note:** * Statistically significant with alpha error = 5%; ** Poor prognosis = composite outcome of death (5) and admission to intensive care unit (3).

## Data Availability

Not applicable.

## LIST OF ABBREVIATIONS

SARS-CoV-2: Syndrome Coronavirus 2
COVID-19: Coronavirus Disease 2019
rRT-PCR: Reverse Transcription Polymerase Chain Reaction
ACTH: Adrenocorticotropic Hormone

## STANDARDS OF REPORTING

STROBE guidelines were followed.
