# Supplementary material for: Predicting Factors of Worse Prognosis in COVID-19: Results from a Cross-sectional Study on 52 Inpatients Admitted to the Internal Medicine Department
Source: Endocr Metab Immune Disord Drug Targets. 2024 Jan 18;24(10):1224–36. doi: 10.2174/0118715303288042240111070057 (PMC11348458; doi:10.2174/0118715303288042240111070057)
Supplement: Supplementary file 1 [file EMIDDT-24-1224_SD1.pdf]

SUPPLEMENTARY MATERIAL

Predicting Factors of Worse Prognosis in COVID-19: Results from a Cross-sectional Study on 52 Inpatients Admitted to the Internal Medicine Department

Giuseppe Lisco[1,*], Antonio Giovanni Solimando[2], Assunta Stragapede[2], Anna De Tullio[1], Cristiana Laraspata[1], Carola Laudadio[1], Vito Angelo Giagulli[1], Marcella Prete[1], Emilio Jirillo[1], Annalisa Saracino[3], Vito Racanelli[4] and Vincenzo Triggiani[1,*]

[1]Interdisciplinary Department of Medicine, Section of Internal Medicine, Geriatrics, Endocrinology and Rare Diseases, School of Medicine, University of Bari Aldo Moro, 70124 Bari, Italy; [2]Guido Baccelli Unit of Internal Medicine, Department of Precision and Regenerative Medicine and Ionian Area-(DiMePRe-J), School of Medicine, Aldo Moro University of Bari, 70124 Bari, Italy; [3]Operative Unit of Infectious Diseases, Hospital-University Polyclinic of Bari, Bari, Italy; [4]Centre for Medical Sciences (CISMed), University of Trento, Trento, Italy

STROBE Statement—Checklist of items that should be included in reports of ***cross-sectional studies***

	Item No	Recommendation	
**Title and abstract**	1	(*a*) Indicate the study's design with a commonly used term in the title or the abstract	pag 1
		(*b*) Provide in the abstract an informative and balanced summary of what was done and what was found	pag 1
**Introduction**			
Background/rationale	2	Explain the scientific background and rationale for the investigation being reported	pag 2
Objectives	3	State specific objectives, including any prespecified hypotheses	pag 2
**Methods**			
Study design	4	Present key elements of study design early in the paper	pag 3
Setting	5	Describe the setting, locations, and relevant dates, including periods of recruitment, exposure, follow-up, and data collection	pag 2
Participants	6	(*a*) Give the eligibility criteria, and the sources and methods of selection of participants	pag 3
Variables	7	Clearly define all outcomes, exposures, predictors, potential confounders, and effect modifiers. Give diagnostic criteria, if applicable	pag 3
Data sources/ measurement	8*	For each variable of interest, give sources of data and details of methods of assessment (measurement). Describe comparability of assessment methods if there is more than one group	pag 4
Bias	9	Describe any efforts to address potential sources of bias	pag 4-5
Study size	10	Explain how the study size was arrived at	na
Quantitative variables	11	Explain how quantitative variables were handled in the analyses. If applicable, describe which groupings were chosen and why	pag 4-5
Statistical methods	12	(*a*) Describe all statistical methods, including those used to control for confounding	pag 4-5
		(*b*) Describe any methods used to examine subgroups and interactions	pag 4-5
		(*c*) Explain how missing data were addressed	pag 4-5
		(*d*) If applicable, describe analytical methods taking account of sampling strategy	pag 4-5
		(*e*) Describe any sensitivity analyses	pag 4-5

**Results**			
Participants	13*	(a) Report numbers of individuals at each stage of study—eg numbers potentially eligible, examined for eligibility, confirmed eligible, included in the study, completing follow-up, and analysed	pag 2
		(b) Give reasons for non-participation at each stage	pag 2
		(c) Consider use of a flow diagram	na
Descriptive data	14*	(a) Give characteristics of study participants (eg demographic, clinical, social) and information on exposures and potential confounders	pag 5 table 1
		(b) Indicate number of participants with missing data for each variable of interest	na
Outcome data	15*	Report numbers of outcome events or summary measures	pag 2
Main results	16	(*a*) Give unadjusted estimates and, if applicable, confounder-adjusted estimates and their precision (eg, 95% confidence interval). Make clear which confounders were adjusted for and why they were included	pag 8-10 table 3 table 7
		(*b*) Report category boundaries when continuous variables were categorized	pag 5
		(*c*) If relevant, consider translating estimates of relative risk into absolute risk for a meaningful time period	na
Other analyses	17	Report other analyses done—eg analyses of subgroups and interactions, and sensitivity analyses	pag 8-10
**Discussion**			
Key results	18	Summarise key results with reference to study objectives	pag 11
Limitations	19	Discuss limitations of the study, taking into account sources of potential bias or imprecision. Discuss both direction and magnitude of any potential bias	pag 11-15
Interpretation	20	Give a cautious overall interpretation of results considering objectives, limitations, multiplicity of analyses, results from similar studies, and other relevant evidence	pag 16
Generalisability	21	Discuss the generalisability (external validity) of the study results	pag 16
**Other information**			
Funding	22	Give the source of funding and the role of the funders for the present study and, if applicable, for the original study on which the present article is based	pag 16

*Give information separately for exposed and unexposed groups.

Note: An Explanation and Elaboration article discusses each checklist item and gives methodological background and published examples of transparent reporting. The STROBE checklist is best used in conjunction with this article (freely available on the Web sites of PLoS Medicine at http://www.plosmedicine.org/, Annals of Internal Medicine at http://www.annals.org/, and Epidemiology at http://www.epidem.com/). Information on the STROBE Initiative is available at www.strobe-statement.org.

Figure 1. Baseline circulating levels of fT_3 in the two examined groups.

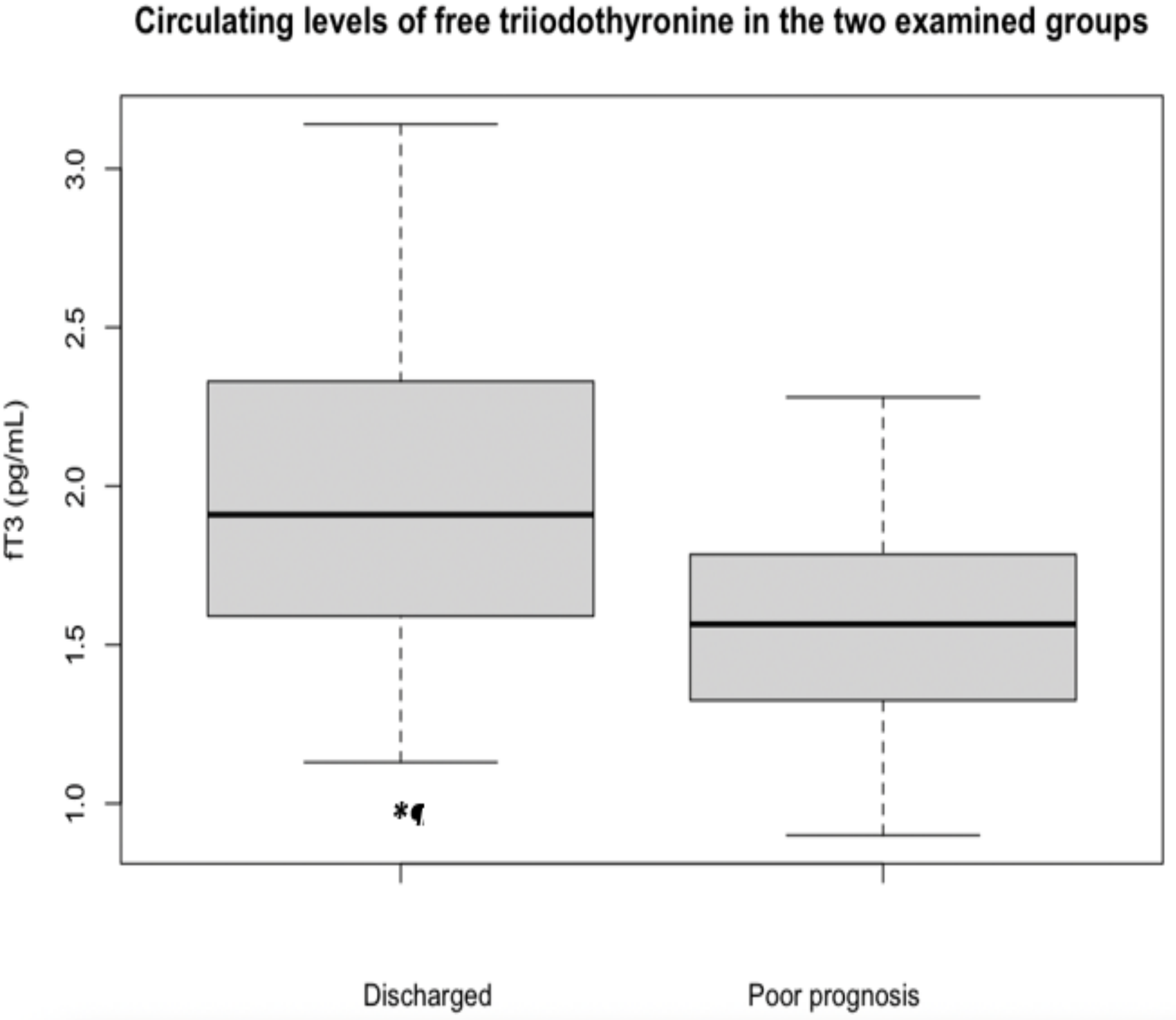

The figure depicts that baseline levels of fT_3 were significantly lower in patients with poor than those with better prognoses (discharged). * p-value <0.05. Poor prognosis = composite outcome of deaths (5) and admissions to intensive care unit (3). Abbreviation: fT_3 = free triiodothyronine.

Figure 2. Baseline circulating levels of IL-6 in the two examined groups.

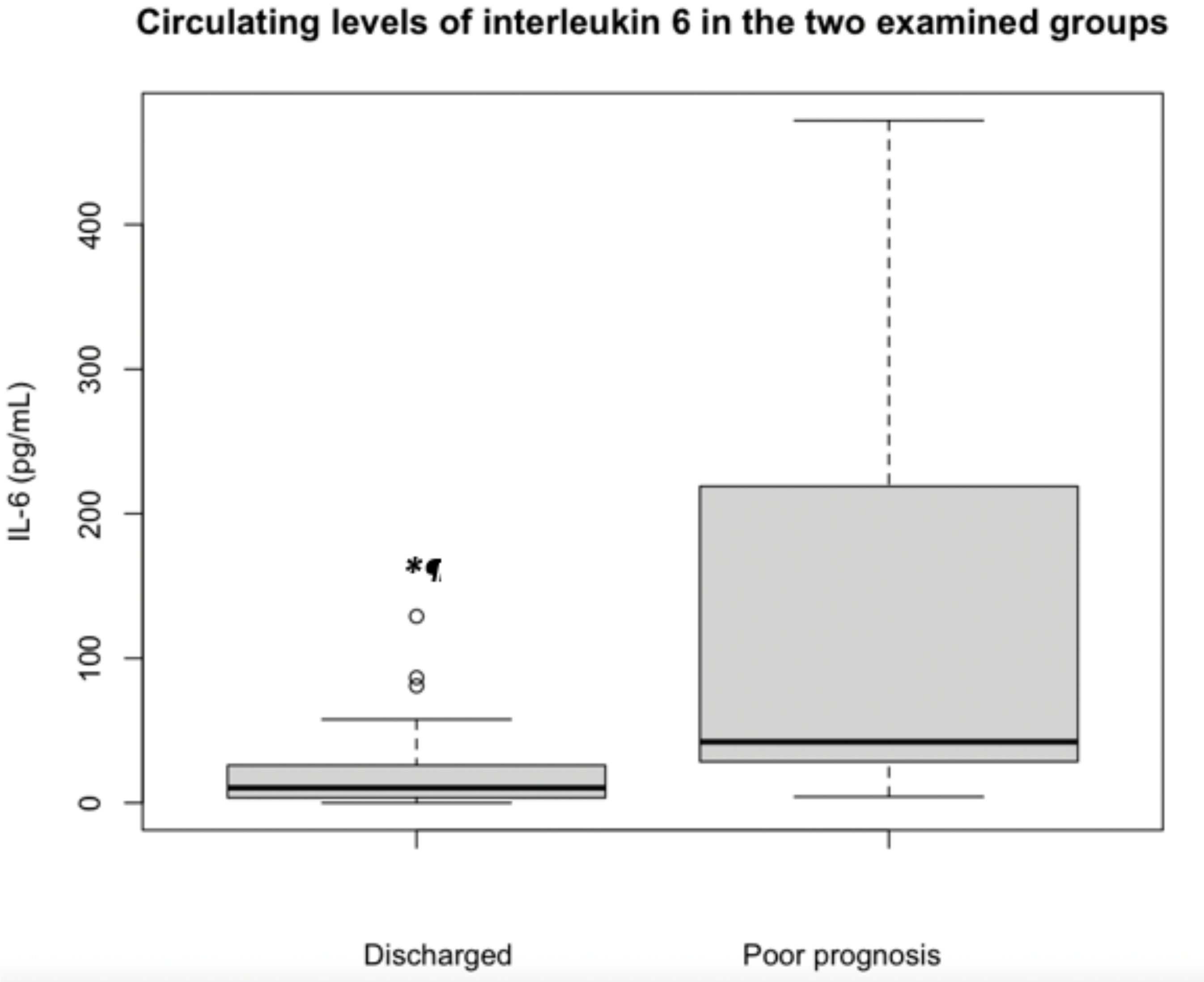

The figure depicts that baseline levels of IL-6 were significantly lower in patients with better (discharged) than those with poor prognoses. * p-value <0.05. Poor prognosis = composite outcome of deaths (5) and admissions to intensive care unit (3). Abbreviation: IL-6 = interleukin 6.

Figure 3. Baseline leukocyte count in the two examined groups.

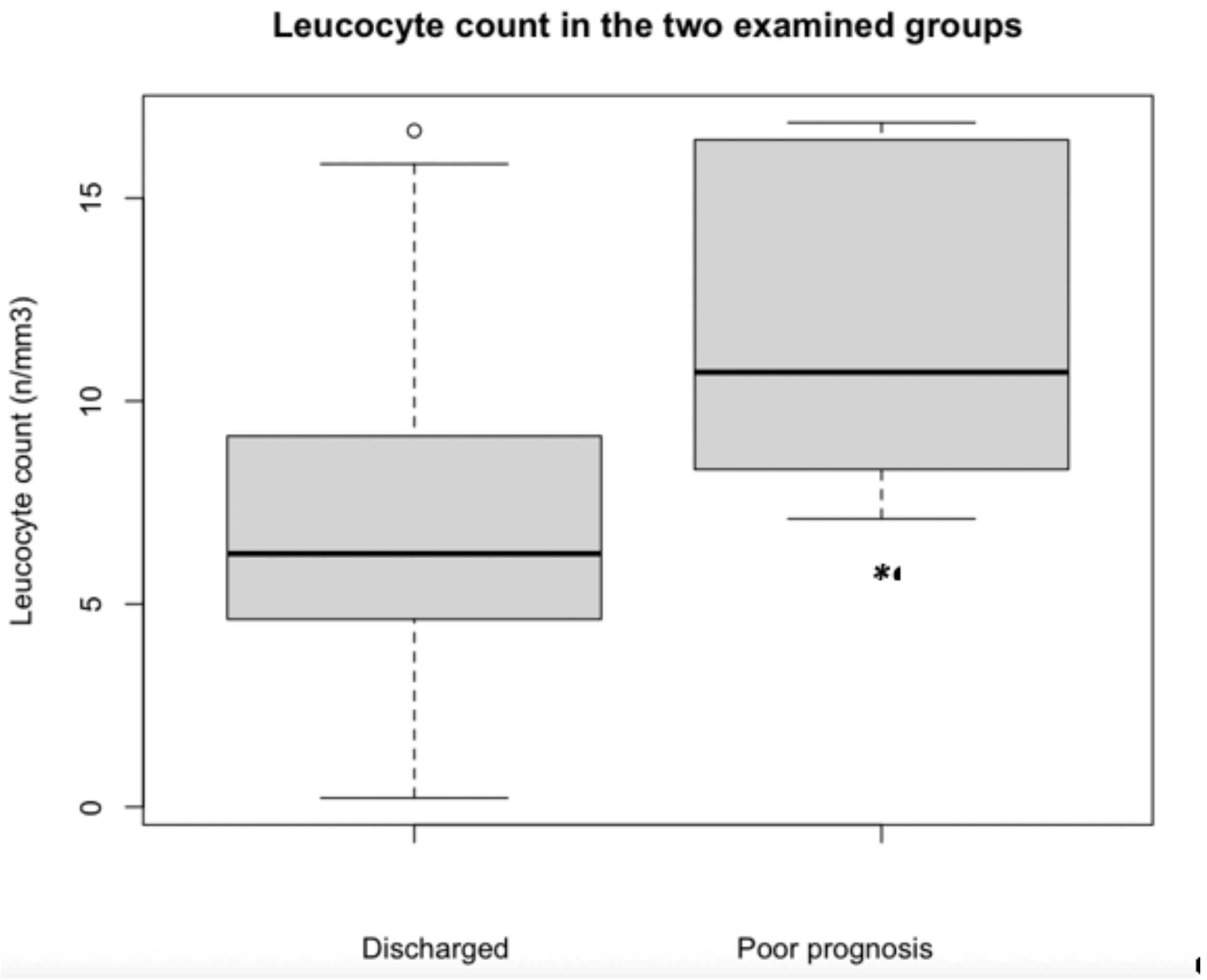

The figure depicts that the baseline leukocyte count was significantly higher in patients with poor than those with better prognoses (discharged). * p-value <0.01. Poor prognosis = composite outcome of deaths (5) and admissions to intensive care unit (3).
